# A novel feedback loop between high MALAT-1 and low miR-200c-3p promotes cell migration and invasion in pancreatic ductal adenocarcinoma and is predictive of poor prognosis

**DOI:** 10.1186/s12885-018-4954-9

**Published:** 2018-10-23

**Authors:** Meng Zhuo, Cuncun Yuan, Ting Han, Jiujie Cui, Feng Jiao, Liwei Wang

**Affiliations:** 1 Department of Oncology, Renji Hospital, School of Medicine, Shanghai Jiaotong University, 160 Pujian Road, Shanghai, 200127 China; 2 Department of Pathology, Eye Ear Nose and Throat Hospital, Fudan University, 83 Fenyang Road, Shanghai, 200031 China

**Keywords:** Pancreatic ductal adenocarcinoma, Metastasis-associated lung adenocarcinoma transcript 1, miR-200c-3p, ZEB1, Feedback loop

## Abstract

**Background:**

It was demonstrated that long non-coding RNAs occupied an important position in tumor pathogenesis and progression. We have previously found that the metastasis-associated lung adenocarcinoma transcript 1 (MALAT-1) promotes cell proliferation and metastases in pancreatic ductal adenocarcinoma (PDAC). The present study was aimed to discuss the underlying mechanisms.

**Methods:**

Bioinformatics method was used to identify the miRNA target of MALAT-1. Expressions of relative genes were assessed by quantitative real-time PCR and western blotting, respectively. Sulforhodamine B assay and Transwell assay were employed to detect cell proliferation, migration and invasion, respectively. Moreover, RNA immunoprecipitation was performed to determine whether RNA-induced silencing complex contained MALAT-1 and its potential binding miRNA. Luciferase assays was used to confirm potential binding site.

**Results:**

Bioinformatics search predicted that miR-200c-3p was a direct target of MALAT-1. Further, we found a reciprocal suppression between MALAT-1 and miR-200c-3p expression. In terms of mechanisms, high MALAT-1 and low miR-200c-3p may form a novel feedback loop. On the one hand, MALAT-1 functioned as a competing endogenous RNA to suppress miR-200c-3p expression, leading to upregulation of ZEB1 expression. On the other hand, miR-200c-3p inhibited the level of MALAT-1 expression was in a way similar to miRNA-mediated downregulation of target genes. Clinical data further indicated that MALAT-1 and ZEB1 expression was negatively correlated with miR-200c-3p transcript level of PDAC tissues. There was a positive correlation between MALAT-1 and ZEB1 level. MALAT-1 (high)/miR-200c-3p (low) correlated with shorter overall survival of PDAC patients. Multivariate analysis revealed that both MALAT-1 and miR-200c-3p levels were independent prognostic factors.

**Conclusion:**

Our findings firstly revealed a novel feedback loop between high MALAT-1 and low miR-200c-3p. Targeting the feedback loop between high MALAT-1 and low miR-200c-3p will be a therapeutic strategy for PDAC.

**Electronic supplementary material:**

The online version of this article (10.1186/s12885-018-4954-9) contains supplementary material, which is available to authorized users.

## Background

Pancreatic ductal adenocarcinoma (PDAC) is considered to be one of the most lethal tumor worldwide [1] and will become the second leading cause of tumor-associated mortality in the USA by 2030 [2]. In spite of rapid progress in understanding PDAC tumorigenesis at the molecular level and therapeutic approaches, the prognosis of PDAC remains poor, with a 5-year survival rate less than 5% [3]. PDAC is insensitive to chemotherapy and radiotherapy, meaning the identification of novel therapeutic targets is imperative.

Non-coding RNAs (ncRNAs) are not transcribed and translated into coding-proteins, including micro-RNAs (miRNAs) and long non-coding RNAs (lncRNAs) [4]. LncRNAs have attracted increasing attention in recent years due to important biological effects in carcinogenesis and progression [5, 6]. In recent years, a large number of lncRNAs, such as MALAT-1, HOTAIR, H19 and PVT1 appear to play important roles in PDAC development, as it regulates cell growth, progression and chemo-resistance, etc. [7]. We have previously found that the MALAT-1 promotes tumor growth and metastases in PDAC [8, 9]. However, the underlying molecular mechanisms still need to be further clarified.

Recently interaction research between lncRNAs and miRNAs is attracting an increasing amount of attention [10]. In this study, we revealed a reciprocal suppression between MALAT-1 and miR-200c-3p expression in PDAC, and performed underlying mechanisms analysis.

## Methods

### Ethics statement

The present study was approved and supervised by the Ethics Committee of the Renji Hospital, School of Medicine, Shanghai Jiaotong University. Written informed consent was obtained from all subjects.

### Patients and samples

Sixty-five paraffin embedded samples were collected from the Surgery Department, Renji Hospital, School of Medicine, Shanghai Jiaotong University from January 2009 to December 2012, and were histopathologically confirmed and staged in accordance with the Union for International Cancer Control. The follow-up data was ended in December, 2017.

### Cell culture

Human pancreatic ductal cells (HPDE) and PDAC cell lines SW1990, CAPAN-1, HS-766 T, CFPAC-1, BxPC-3, AsPC-1 and PANC-1 were all obtained from Chinese Academy of Sciences Cell Bank (Shanghai, China). SW1990 cell was cultured in L-15 medium supplemented with 10% fetal bovine serum (FBS, all purchased from Gibco, Grand Island, NY, USA), and grown in room temperature air. The other cells were cultured in RPMI 1640 supplemented with 10% FBS (all purchased from Gibco, Grand Island, NY, USA), grown at 37 °C in 5% CO_2_ saturated humidity.

### RNA extraction and reverse transcription quantitative real-time polymerase chain reaction (RT-qPCR)

The miRNeasy FFPE Kit (QIAGEN, Hilden, Germany) kit and miniBEST universal RNA extraction kit (Takara Bio, Dalian, China) was used to extract total RNA from tissue samples and cultured cells, respectively. Reverse transcription and RT-qPCR kits were employed to examine MALAT-1 and miR-200c-3p level. RT-qPCR reactions were performed by the ViiATM 7 system (Applied Biosystems Inc. Foster City, CA, USA). The primers used in the reaction for MALAT-1, miR-200c-3p, ZEB1, GAPDH and U6 were purchased from Tiangen (Tiangen, Beijing, China). GAPDH and U6 were detected as the endogenous control to normalize the expression levels of the different genes. Comparative threshold cycle (Ct) (2-^ΔΔCt^) method was selected to calculate relative expression.

### Cell proliferation assay

Cell proliferation was measured via the sulforhodamine B assay. Cells were seeded in 96-well plates and transfected with miR-200c-3p nc, miR-200c-3p inhibitor, or mimics and cultured for 24, 48, 72, and 96 h. Using microtiter plate reader (VERSMax), optical density (OD) values were measured at 560 nm.

### Cell migration and invasion assays

Invasion assays were conducted 24 h after transfection. Briefly, 1 × 10^5^ cells in serum-free media were seeded into the upper chamber of an insert (8.0 μm, Millipore, Billerica, MA, USA) that was coated with Matrigel (BD Biosciences, Franklin Lakes, NJ, USA) [9]. Then, 10% FBS-containing medium was placed in the lower chambers of the insert. After incubation for 24 h, the invaded cells were fixed and stained using 0.1% crystal violet staining solution. Finally, the cells were counted by a microscope at a magnification of × 200 in five randomly selected fields. Migration assays were similar to the above-mentioned assay but no Matrigel was used.

### RNA binding protein immunoprecipitation (RIP)

RIP assay was performed by the Magna RIP™RNA-Binding Protein Immunoprecipitation Kit (Millipore, Billerica, MA, USA). Cells were lysed in RIP Lysis Buffer. MiR-200c-3p and MALAT-1 were immunoprecipitated with AGO2 antibodies. The complexes that bound on the magnetic beads were immobilized and unbound materials were washed off. Then, miR-200c-3p and MALAT-1 were extracted and analyzed by RT-qPCR.

### Luciferase assays

Firstly, 293 T cells were seeded in 24-well plates and transfected with 50 nM miR-200c-3p mimics or miR-200c-3p nc respectively. Secondly, after 24 h transfection, the above 293 T cells were then transfected with 100 ng luciferase reporter vectors that contained either wild-type (MALAT-1-wt) or mutant types (MALAT-1-mut) (both were designed and structured from Obio Technology, Shanghai, Corp. Ltd). The Renilla luciferase reference plasmid was included in each transfection system as an internal control to normalize the transfection efficiency. Finally, at 48 h after transfection, the Dual-Luciferase Reporter Assay using an illuminometers (Promega Corporation, Madison, WI) was performed according to the manufacturer’s protocol.

### Western blot analysis

Western blot analysis was conducted as previously described [11]. The primary antibodies were as follows: anti-AGO2 (1:1000), anti-ZEB1 (1:1000, Cell Signaling Technology, Beverly, MA, USA), and anti-β-Actin (1:2500, Santa Cruz Biotechnology, Santa Cruz, CA, USA).

### Plasmid construction and transfection

Recombinant plasmid pcDNA3.0/ZEB1, miR-200c mimic, miR-200c inhibitor, siRNA against MALAT-1, and negative controls were all obtained or constructed from GenePharma (Shanghai, China). The detail sequences were under Additional file 1. Lipofectamine 2000 (Invitrogen, USA) was used as transfections agents.

### Statistical analyses

Data were presented as the mean ± SE. All statistical analyses were conducted by SPSS statistical software 17.0 (SPSS Inc., Chicago, IL, USA). For comparison between two groups, we used two-tailed Student’s t test. Multiple group comparisons were calculated with one-way ANOVA analysis. Least-significant difference (LSD) was used for post hoc test. χ2 tests or Fisher’s exact methods was used to detect correlation between MALAT-1 level or miR-200c-3p and clinical characteristics, as appropriate. The correlation between MALAT-1, miR-200c-3p and ZEB1 expression was analyzed using Pearson Correlation. Kaplan-Meier method was used to estimate overall survival (OS). Univariate and multivariate COX regression analysis was performed. *P* < 0.05 was considered to indicate a statistically significant difference.

## Results

### Elevated level of MALAT-1 inhibits miR-200c-3p expression in PDAC

**Fig. 1:**
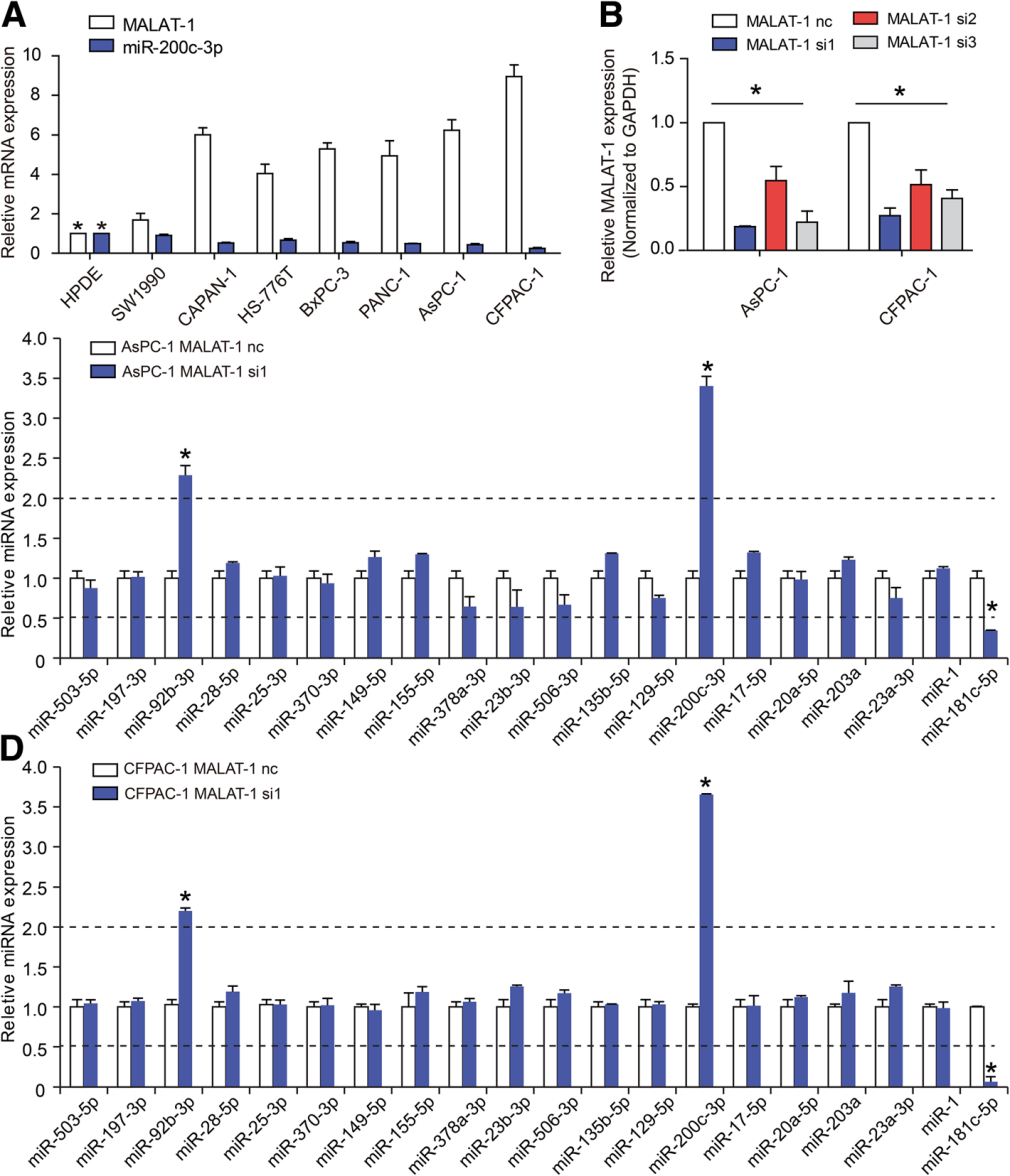
Elevated level of MALAT-1 inhibits miR-200c-3p expression in PDAC. **a** High level of MALAT-1 and low level of miR-200c-3p were expressed in PDAC cell lines compared to HPDE. **b** RT-qPCR was employ to detect the efficiency of MALAT-1 knockdown in AsPC-1 cell line. **c, d** Twenty miRNAs candidate were chosen to validate in response to knockdown of MALAT-1 in AsPC-1 (**c**) and CFPAC-1 (**d**) cell lines. Initial profiling identified three miRNAs, including miR-200c-3p, miR-92b-3p, and miR-181c-5p, that exhibited > 2-fold-change compared with the control, and miR-200c-3p showed the greatest fold-change in response to the MALAT-1 knockdown

To investigate the interaction among potential miRNAs and MALAT-1, we used the Starbase v2.0 (starbase.sysu.edu.cn LncRNA.php) to search for miRNAs that complementarily base-pair with MALAT-1. Twenty miRNAs were predicted to bind to MALAT-1 (see Additional file 2). We further validated 20 miRNAs expression after knockdown of MALAT-1 in CFPAC-1 and AsPC-1 cell lines, which showed relative high level of MALAT-1 (Fig. 1a and b). Initial profiling identified three miRNAs (miR-200c-3p, miR-92b-3p, and miR-181c-5p) that exhibited > 2-fold-change compared with the control (Fig. 1c and d; *P* < 0.05). Next, miR-200c-3p was chosen for the study because it showed the greatest fold-change in response to the MALAT-1 knockdown.

### Anti-miR-200c-3p restores MALAT-1-siRNA function

**Fig. 2:**
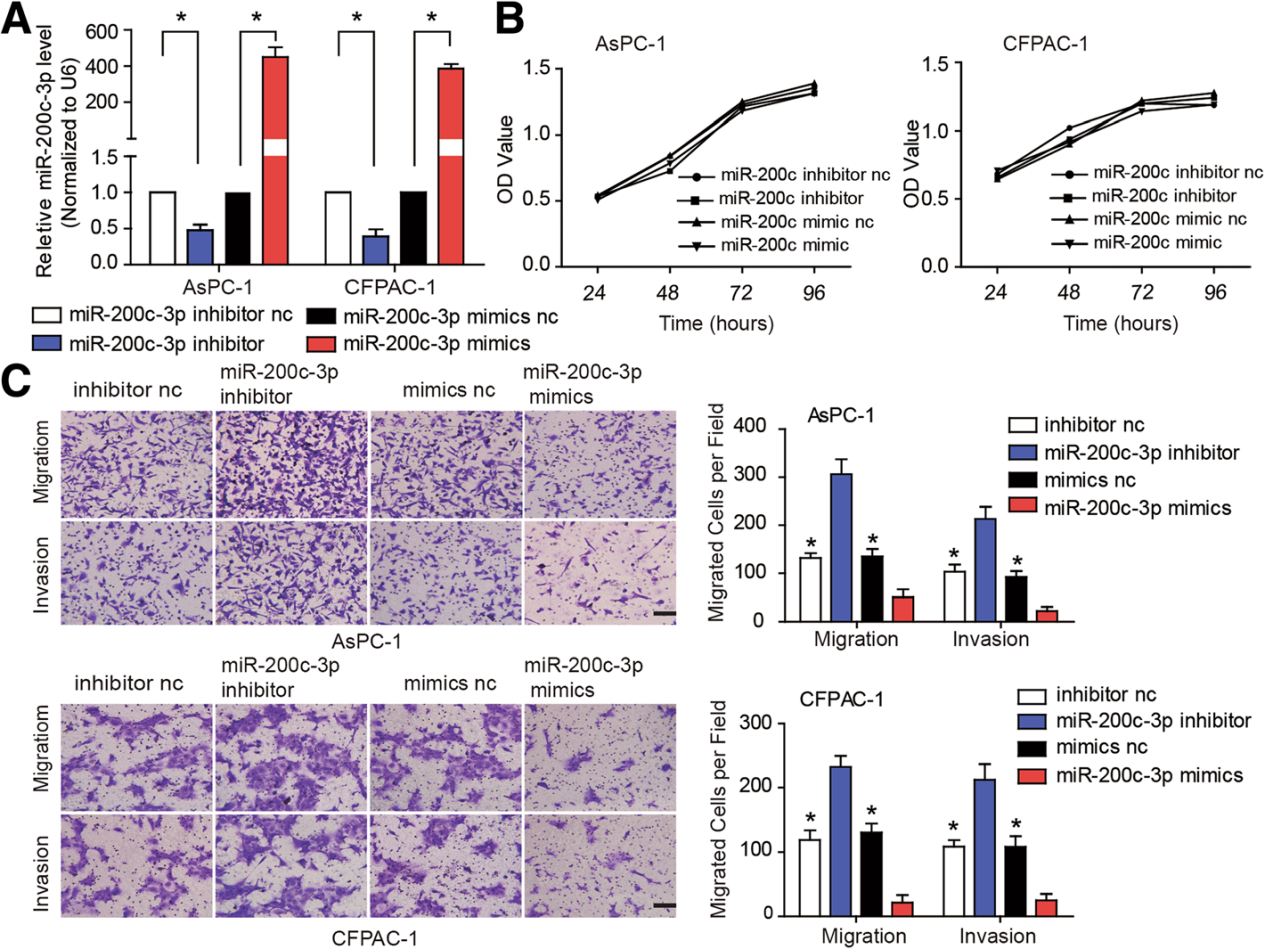
MiR-200c-3p suppress cell migration and invasion, but not cell proliferation in PDAC. **a** RT-qPCR was employ to detect the transfection efficiency of miR-200c-3p nc, inhibitors, or mimics. **b** Sulforhodamine B assay was used to detect cell proliferation ability in different groups, including miR-200c-3p nc, inhibitors, or mimics group. The cell proliferation ability had no differences **c.** A transwell migration assay to examine the effect of miR-200c-3p on PDAC cell migration and invasion. Transfection of miR-200c-3p mimics reduced the number of migrating cells as compared to that of miR-200c-3p nc, while transfection of miR-200c-3p inhibitor increased the number of migrating cells

Subsequently, to study the biological function of miR-200c-3p on PDAC, we transfected AsPC-1 and CFPAC-1 cells with miR-200c-3p nc, inhibitors, or mimics (Fig. 2a; *P* < 0.05). Sulforhodamine B assay showed that there were no differences of cell proliferation ability in different groups, including miR-200c-3p nc, inhibitors, or mimics group (Fig. 2b; *P* < 0.05). Transwell assay revealed that miR-200c-3p mimics transfection decreased the number of migrating cells in comparisons with that of miR-200c-3p nc, while transfection of miR-200c-3p inhibitor increased the number of migrating cells (Fig. 2c; *P* < 0.05). Taken together, miR-200c-3p suppressed cell migration and invasion in PDAC, but not cell proliferation.

**Fig. 3:**
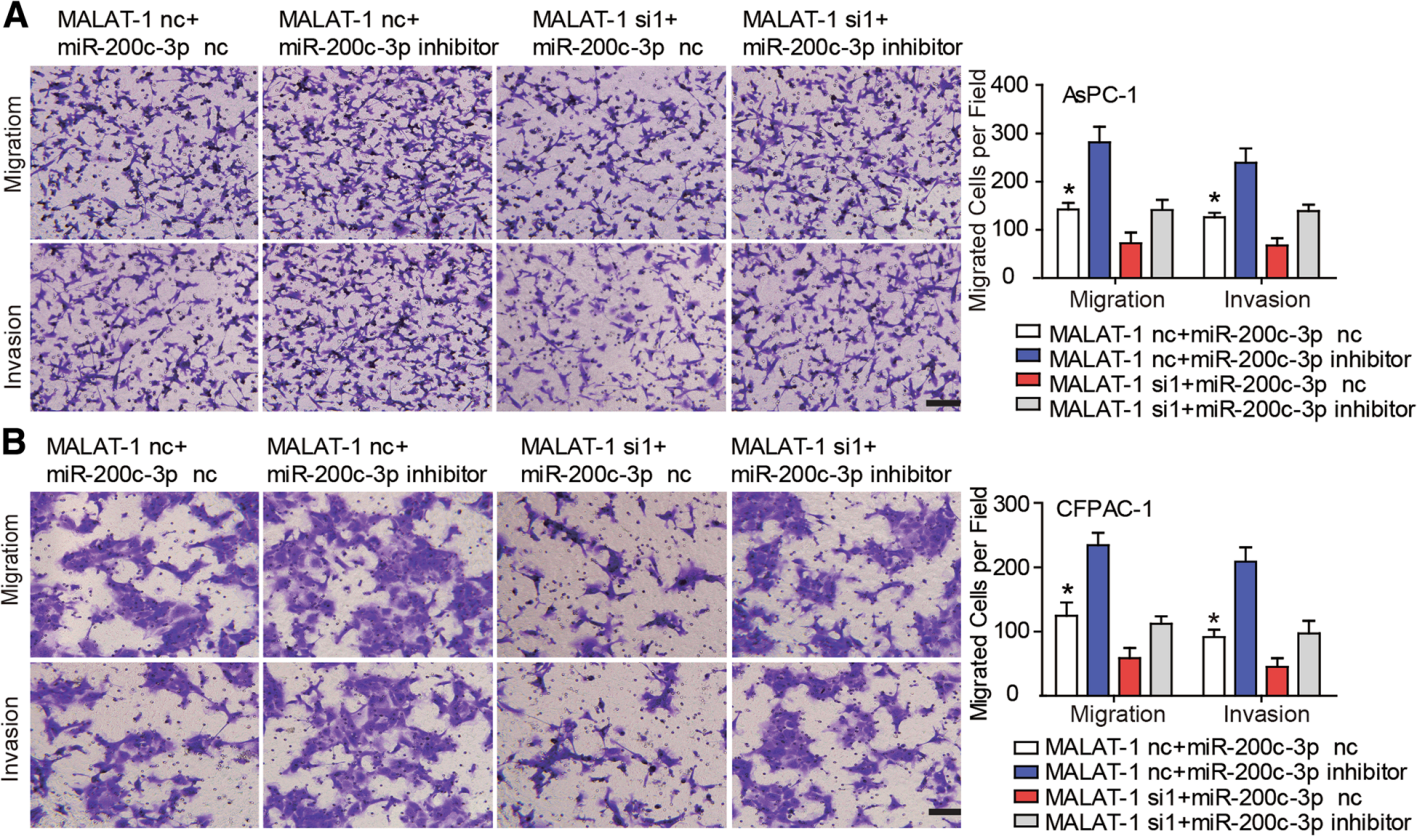
Anti-miR-200c-3p restores MALAT-1-siRNA function. **a, b** Transwell migration assays were employed to detect cell migration ability when co-transfected AsPC-1 (**a**) and CFPAC-1 (**b**) cells with MALAT-1 siRNA and miR-200c inhibitor. The results demonstrated that MALAT-1 knockdown inhibited the migration and invasion of pancreatic cancer cells, whereas miR-200c-3p inhibition partially reversed decreased invasiveness

Our previous studies have revealed that MALAT-1 promotes tumor growth and metastases in PDAC [8, 9]. To further examine if the effects of MALAT-1 on cell migration and invasion were partially mediated by miR-200c-3p, we co-transfected CFPAC-1 and AsPC-1 cells with MALAT-1 siRNA and miR-200c-3p inhibitor. The results revealed that knockdown of MALAT-1 suppressed cell migration and invasion, whereas miR-200c-3p inhibition partially reversed decreased invasiveness (Fig. 3a and b; *P* < 0.05). These findings were also confirmed in PANC-1 and CAPAN-1 cell lines (see Additional file 3; *P* < 0.05). Together, the data suggested that miR-200c-3p can partially rescue the loss of MALAT-1-mediated PDAC cell migration and invasion.

### MALAT-1 upregulates ZEB1 expression by sponging miR-200c-3p

**Fig. 4:**
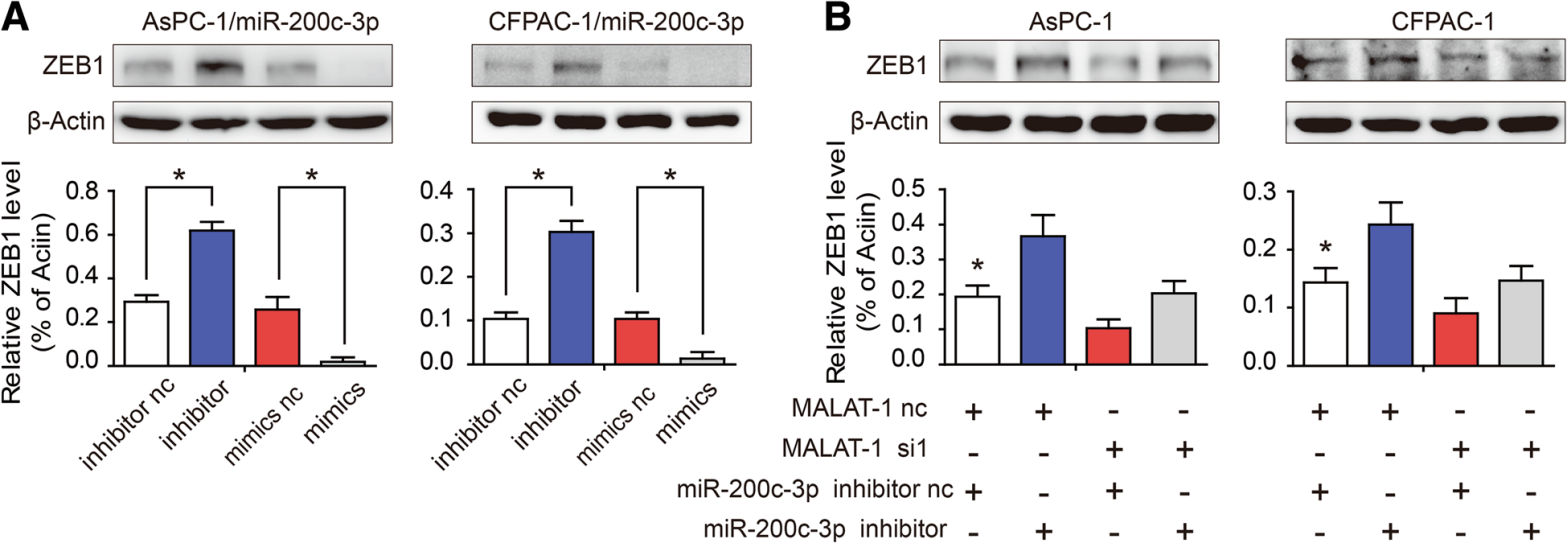
MALAT-1 upregulates ZEB1 expression by sponging miR-200c-3p. **a** The protein levels of ZEB1 were detected in response to transfection of miR-200c-3p nc, inhibitors, or mimics. The result showed that transfection of miR-200c-3p inhibitor triggered significant endogenous ZEB1 expression compared to that of nc in AsPC-1 and CFPAC-1 cells, whereas miR-200c-3p mimic silenced ZEB1 protein expression. **b** Western blot was performed to examine ZEB1 protein expression in Aspc-1 and CFPAC-1 after co-transfection with MALAT-1 nc/si1 or miR-200c-3p nc/inhibitor. Cotransfection with MALAT-1 siRNA and miR-200c-3p inhibitor showed that the miR-200c-3p inhibitor weakened the down-regulation of ZEB1 by MALAT-1 knockdown. β-actin was used as an internal control for protein loading. Relative densities are presented as the fold change relative to the internal control

**Fig. 5:**
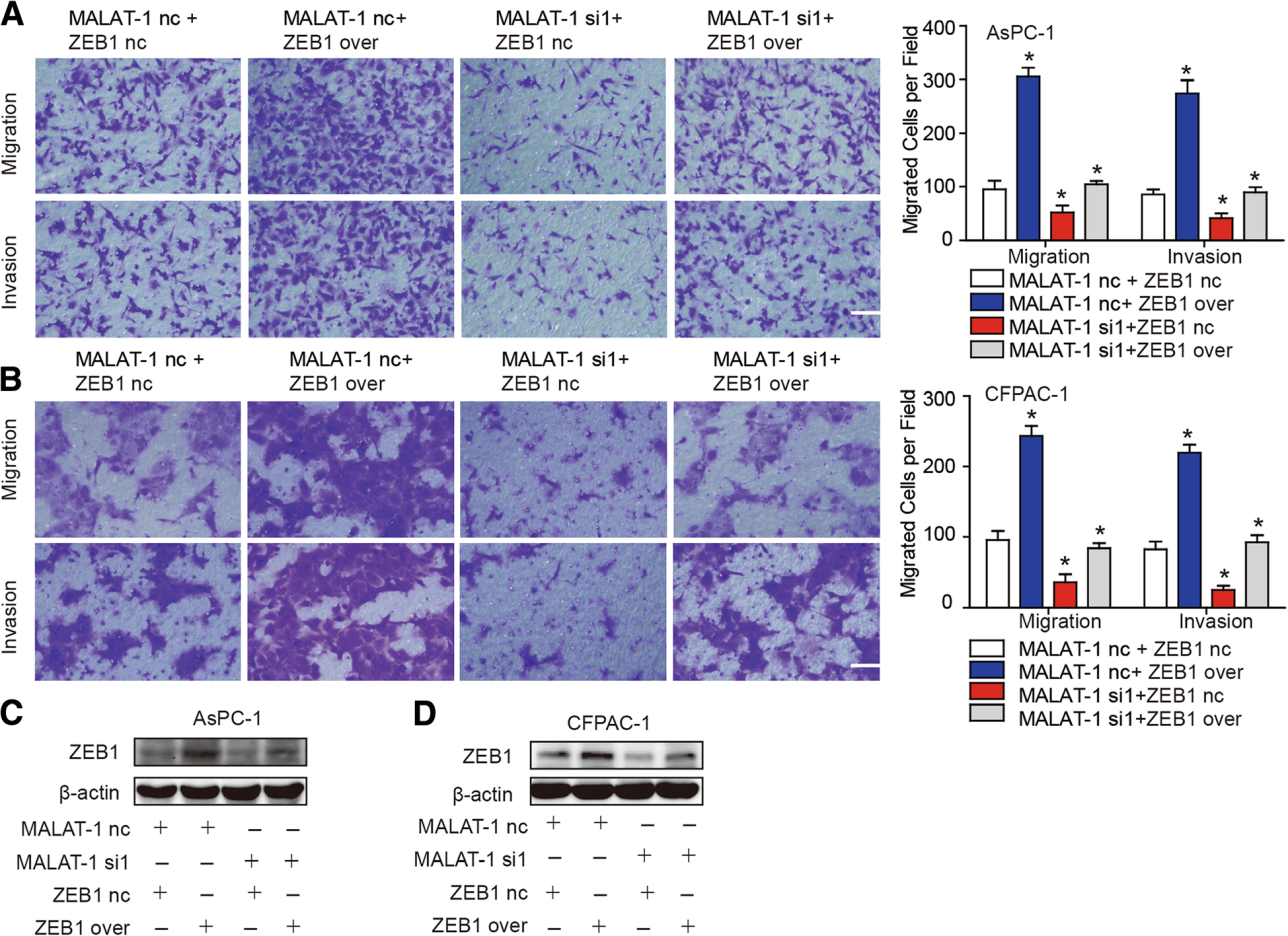
ZEB1 overexpression rescue the loss of MALAT-1-mediated reduced cell migration and invasion. **a, b** Transwell migration assays were employed to detect cell migration ability when co-transfected AsPC-1 (**a**) and CFPAC-1 (**b**) cells with MALAT-1 nc/siRNA and ZEB1 nc/over. **c, d** Western blot was performed to examine ZEB1 protein expression in Aspc-1 and CFPAC-1 after co-transfection with MALAT-1 nc/siRNA and ZEB1 nc/over

Among the many targets of miR-200c-3p, we focused on ZEB1 because it encodes a zinc finger and homeodomain transcription factor proteins that function in PDAC metastasis. Firstly, the result verified that the miR-200c-3p inhibitor triggered significant endogenous ZEB1 expression, whereas miR-200c-3p overexpression silenced ZEB1 protein expression (Fig. 4a; *P* < 0.05). Furthermore, cotransfection with MALAT-1 siRNA and miR-200c-3p inhibitor revealed that the miR-200c-3p inhibitor weakened down-regulation of ZEB1 by MALAT-1 knockdown (Fig. 4b; *P* < 0.05). Further study showed that ZEB1 overexpression can rescue the loss of MALAT-1-mediated repression activity in PDAC, including reduced cell migration and invasion (Fig. 5a, b, c and d; *P* < 0.05). This experimental evidence supported that the observed migration/invasion defect in MALAT-1 knockdown or miR200c-3p mimics was in fact mediated via regulation of ZEB1 expression.

### MALAT-1 was a target of miR-200c-3p

**Fig. 6:**
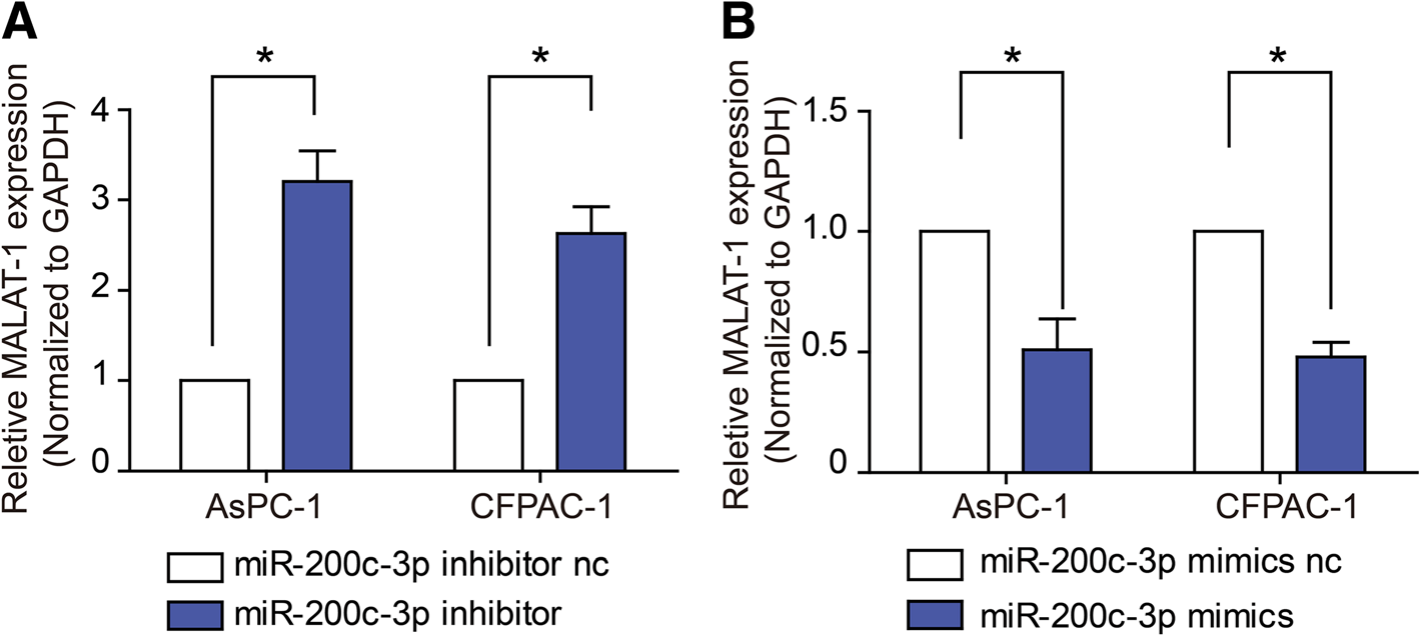
MALAT-1 was a target of miR-200c-3p. **a, b** RT-qPCR was used to measure MALAT-1 expression levels in response to transfection of miR-200c-3p nc, mimic or inhibitor. The results showed that inhibiting miR-200c-3p up-regulated MALAT-1 levels (**a**), whereas miR-200c-3p overexpression significantly decreased MALAT-1 in both cell lines (**b**)

We then measured MALAT-1 expression levels in response to transfection of miR-200c-3p mimic or inhibitor. The results showed that inhibiting miR-200c-3p up-regulated MALAT-1 levels (Fig. 6a; *P* < 0.05), whereas miR-200c-3p overexpression significantly decreased MALAT-1 in both cell lines (Fig. 6b; *P* < 0.05). The data above implicated MALALT-1 was also a target of miR-200c-3p.

### MALAT-1 and miR-200c-3p are associated with immunoprecipitated AGO2 complex

**Fig. 7:**
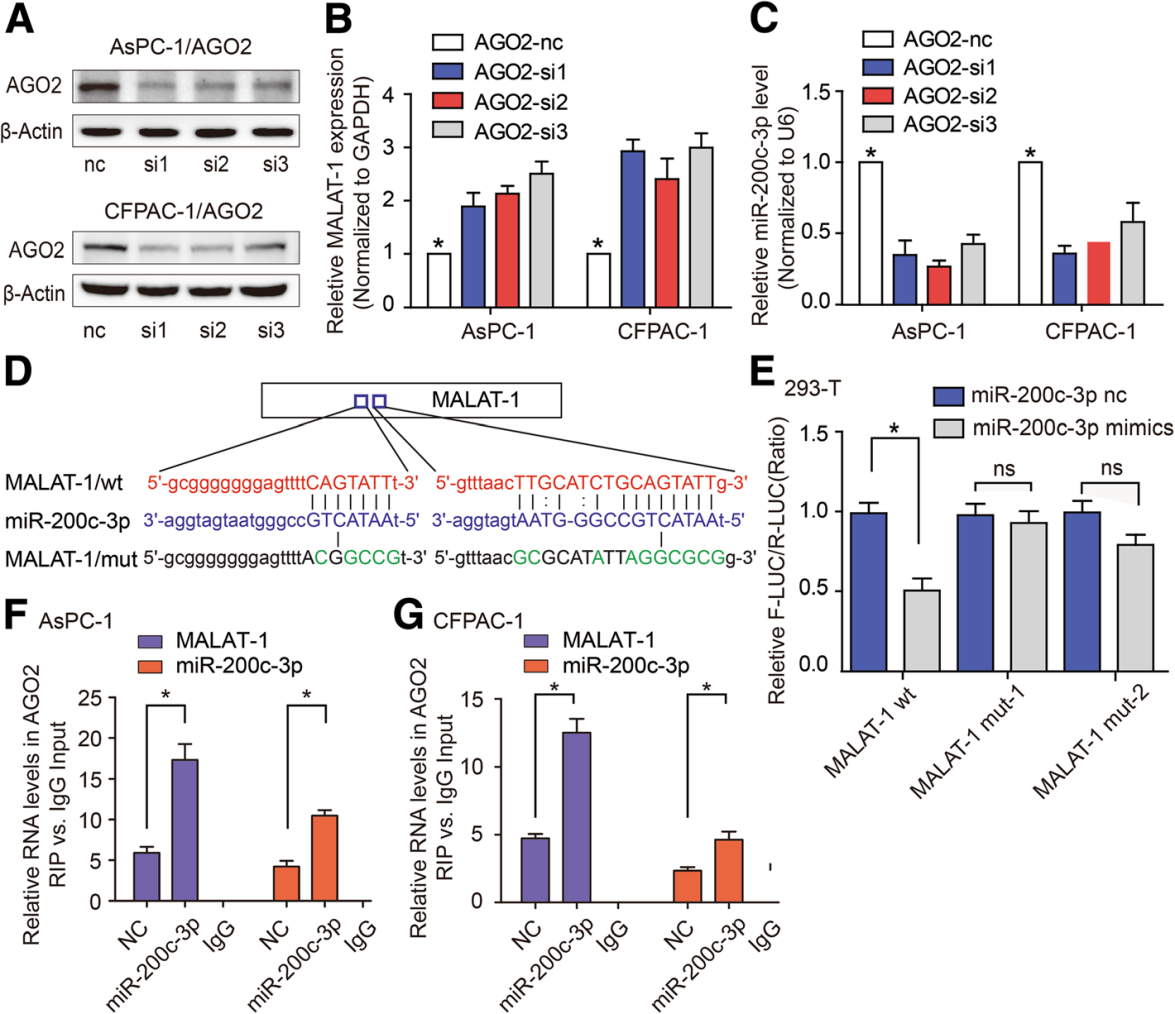
MALAT-1 and miR-200c-3p are associated with the immunoprecipitated AGO2 complex. **a** Western blot was employed to detect AGO2 expression after AGO2 knockdown in AsPC-1 and CFPAC-1 cells. **b, c** RT-qPCR was use to examine MALAT-1 (**b**) and miR-200c-3p (**c**) level in response to knockdown of AGO2 in AsPC-1 and CFPAC-1, respectively. **d** Binding sites for miR-200c-3p in MALAT-1, as predicted by Starbase v2.0. The parts in the column imply the possible binding sites in MALAT-1. **e** Seed matches from MALAT-1 wt or its mutants (MALAT-1-mut-1 and MALAT-1-mut-2) that were devoid of specific miR-200c-3p-binding sites were mutagenized and transfected into 293 T cells together with 50 nM of miR-200c-3p mimics or nc. MiR-200-3p mimics significantly decreased luciferase activity only in the MALAT-1-wt construct. **f, g** RIP with monoclonal anti-Ago2 and IgG from AsPC-1 (**f**) and CFPAC-1 (**g**) cell extracts transfected with nc or miR-200c-3p mimics. Levels of MALAT-1 and miR-200c-3p were measured by RT-qPCR

AGO2 is necessary for miRNA-mediated gene silencing [12]. To investigate whether MALAT-1 expression is controlled by miR-200c-3p, we knocked down AGO2 in AsPC-1 and CFPAC-1 (Fig. 7a; *P* < 0.05). As expected, MALAT-1 expression was increased in AGO2-knockdown cells (Fig. 7b; *P* < 0.05). Also, the stability of miR-200c-3p was significantly impaired in AGO2-knockdown cells (Fig. 7c; *P* < 0.05). These data collectively suggest that miR-200c-3p directly regulates MALAT-1 levels.

To further study the mechanism that underlies the negative regulation of miR-200c-3p by MALAT-1 and to obtain evidence that supports a potential interaction between these two genes, we used Starbase v2.0 to predict the interaction sites between miR-200c-3p and MALAT-1 (Fig. 7d), and cloned the two mutated binding sites to vector, respectively. The mutants were designated as pMALAT-1-mut1 and pMALAT-1-mut2 (Fig. 7d). Then, luciferase assays were performed in 293FT cells that were transfected with pMALAT-1-mut1 or pMALAT-1-mut2. The results showed that luciferase activity decreased in the pMALAT-1 WT group in 293 T-transfected miR-200c-3p mimics compared to control group (Fig. 7e; *P* < 0.05). However, there were no effects on the luciferase reporter activities of pMALAT-1 mut-1 and pMALAT-1 mut-2 (Fig. 7e; *P* < 0.05). Collectively, these results suggested that MALAT-1 was a direct target gene of miR-200c. Further, AGO2 RIP assay showed that transfection with miR-200c-3p mimics enriched MALAT-1 and miR-200c-3p in AGO2 immunoprecipitated from AsPC-1 (Fig. 7f; *P* < 0.05) and CFPAC-1 (Fig. 7g; *P* < 0.05) cell extracts. Collectively, these results were consistent with the hypothesis that MALAT-1 stability is AGO2-dependent and is regulated by miR-200c-3p in PDAC.

### MALAT-1 expression is negatively associated with miR-200c-3p in PDAC tissues

**Fig. 8:**
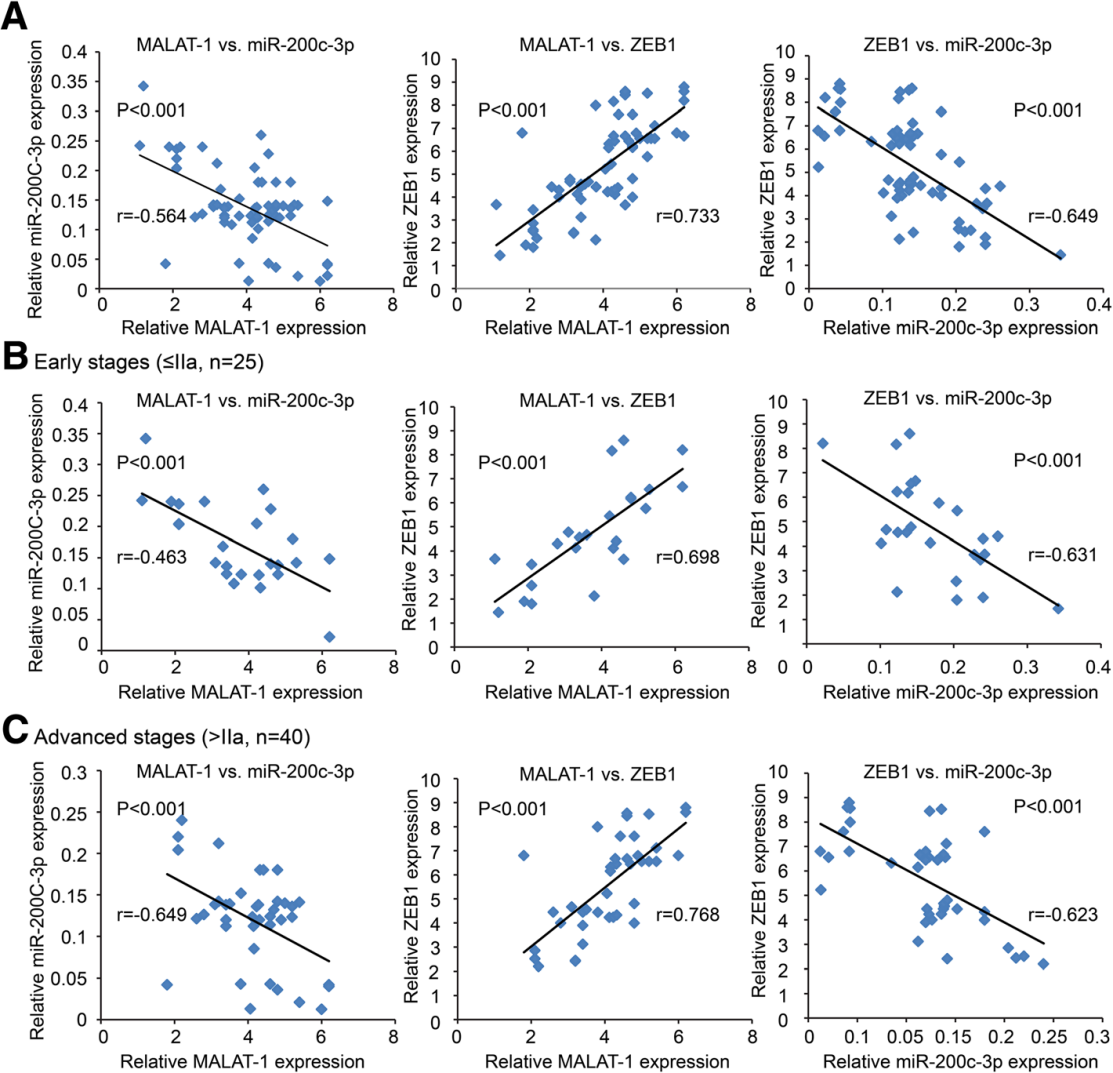
Correlation between MALAT-1, miR-200c-3p and ZEB1 expression in PDAC patients. **a** Pearson correlation analysis showed that expression of MALAT-1 and ZEB1 was negatively correlated with miR-200c-3p transcript level. And there was a positive correlation between MALAT-1 and ZEB1 level. **b, c** The phenomenon of correlation between MALAT-1, miR-200c-3p and ZEB1 were consistent at different clinical stages, including early (≤ II**a**, **b**) and advanced stage (> II**a**, **c**)

We further evaluated the correlation between MALAT-1 and miR-200c-3p expression and the clinicopathological characteristics. The median value was used as cut-off to divide MALAT-1 or miR-200c-3p expression into low level group (*n* = 33) and high level group (*n* = 32). As shown in Additional file 4, MALAT-1 expression is strongly correlated to lymph node metastasis (*P* = 0.007) and clinical stage (*P* = 0.007). And miR-200c-3p expression was negatively correlated with gender (*P* = 0.011), lymph node metastasis (*P* = 0.017), and clinical stage (*P* = 0.017). Bivariate correlation analysis showed that expression of MALAT-1 and ZEB1 was negatively correlated with miR-200c-3p transcript level of PDAC tissues (Fig. 8a; *P* < 0.05). There was a positive correlation between MALAT-1 and ZEB1 level (Fig. 8a; *P* < 0.05). And this phenomenon was consistent at different clinical stages (Fig. 8b and c; *P* < 0.05).

### Association between MALAT-1 and miR-200c-3p expression and prognosis in PDAC patients

**Fig. 9:**
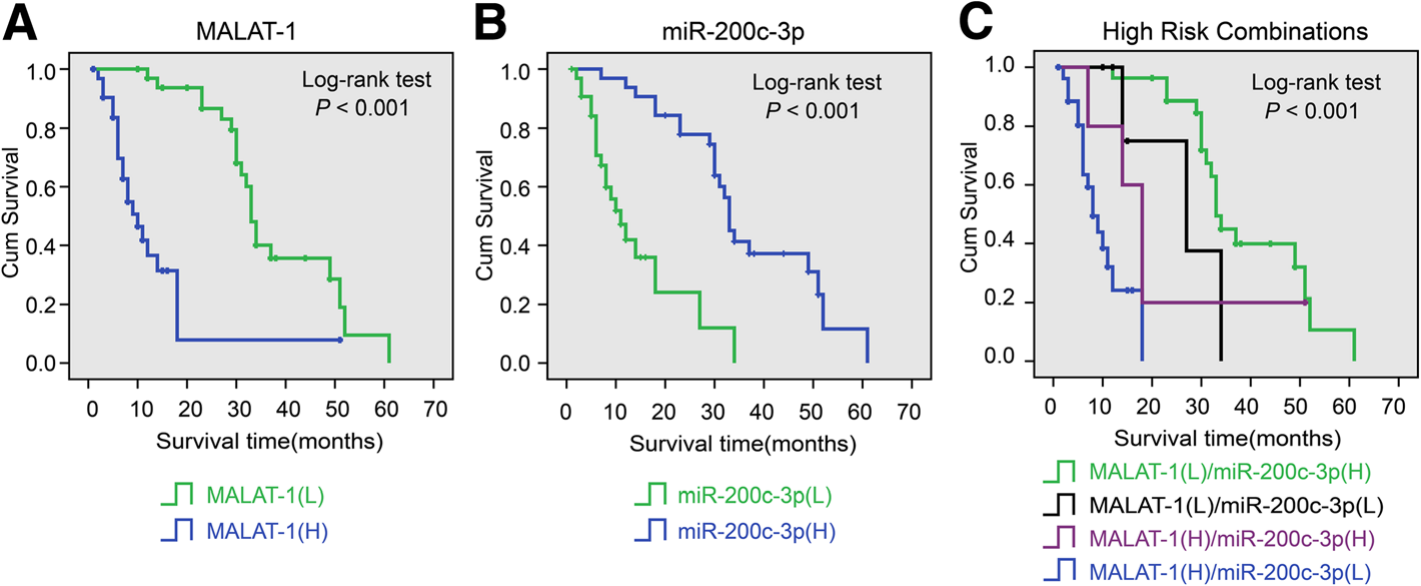
Association between MALAT-1 and miR-200c-3p expression and prognosis in PDAC patients. **a** The log-rank test showed that patients with PDAC tumors expressing high MALAT-1 levels had significantly lower OS than those expressing low MALAT-1. **b** The patients expressing high miR-200c-3p levels had significantly higher OS than those expressing low miR-200c-3p. **c** The log-rank test showed that MALAT-1 (high)/ miR-200c-3p (low) correlated with shorter OS of PDAC patients compare to that of other groups, including MALAT-1 (low)/miR-200c-3p (low), MALAT-1 (low)/ miR-200c-3p (high) and MALAT-1 (high)/ miR-200c-3p (high)

Kaplan-Meier analysis showed that patients with PDAC tumors expressing high MALAT-1 levels had significantly lower OS than those expressing low MALAT-1 (Fig. 9a; *P* < 0.001), whereas that patients expressing high miR-200c-3p levels had significantly higher OS than those expressing low miR-200c-3p (Fig. 9b; *P* < 0.001). To further investigate the correlation of OS with MALAT-1 and miR-200c-3p expression, the results showed that MALAT-1 (high)/ miR-200c-3p (low) associated with shorter OS of PDAC patients (Fig. 9c; *P* < 0.001).

Using univariate analysis, in addition to clinical stage, both MALAT-1 (*P* < 0.001) and miR-200c-3p (*P* < 0.001) levels were closely associated with OS (see Additional file 5). Multivariate analysis revealed that both MALAT-1 (*P* < 0.001) and miR-200c-3p (*P* < 0.001) level was an independent prognostic factor for PDAC patients (see Additional file 5).

## Discussion

Gathering evidence demonstrated that lncRNAs occupies an important position in cancer pathogenesis [4, 13]. Previously, we have outlined the functional lncRNAs in PDAC, such as MALAT-1, HOTAIR, H19 and HULC, and decipher possible mechanisms of lncRNAs [7]. MALAT-1, one of the first lncRNAs, was demonstrated to be associated with lung cancer [14]. Also, MALAT-1 has been linked to several other human tumor entities [15, 16]. In our previous studies, we have found that the MALAT-1 served as oncogenic lncRNA participating in PDAC cell growth and progression [8, 9]. However, the underlying molecular mechanisms are far from being fully elucidated.

Amounting studies indicate that MALAT-1 could exert functions by targeting miRNAs. Han X et al. [17] found that MALAT-1 modulated Srf through miR-133 and discovered a novel correlation among MALAT-1, miR-133, and Srf in myoblast differentiation. Lei R et al. [18] revealed that high level of MALAT-1 promoted cell growth by targeting miR-506 in ovarian cancer. In our study, we found that MALAT-1 knockdown decreased the expression of miR-200c-3p in PDAC. On the other hand, miR-200c-3p mimic decreased the level of MALAT-1, while miR-200c-3p inhibitor upregulated MALAT-1 level. A luciferase assays further confirmed that MALAT-1 was a direct target of miR-200c-3p in PDAC.

Subsequently, we discussed the mechanism of such a feedback loop. It was found that MALAT-1 and miR-200c-3p bind to the same RNA-induced silencing complex. Based on the fact that lncRNAs act as ceRNA to bind specific miRNAs and regulate their function [19, 20]. We supposed that MALAT-1 may inhibit miR-200c-3p expression in such way in PDAC. It was found that MALAT-1 could upregulate ZEB1 expression, which was a target of miR-200c-3p. Together, the above results further confirm our hypothesis. For another, miRNAs regulate protein-coding gene expression at transcriptional level through binding to the 3′-untranslated regions [21, 22]. Leucci E et al. [23] found that miR-9 targeted the AGO2-mediated deregulation of MALAT-1 in the nucleus. Wang X et al. [24] showed that posttranscriptional inhibitory mechanism of MALAT-1 by miR-101 and miR-217 suppressed cell proliferation, migration, and invasion in esophageal squamous cell carcinoma. We proposed that the way that miR-200c-3p inhibited the level of MALAT-1 expression is in a way similar to miRNA-mediated silencing of protein-coding genes. Our data were consistent with recent report in ovarian cancer [12]. Hirata H et al. [25] found that MALAT-1 promoted aggressive phenotype by interacts with miR-205 and EZH2 in renal cell carcinoma. Our previous study had demonstrated that MALAT-1 could recruit EZH2 to the E-cadherin promoter, where it repressed E-cadherin expression, leading to cell migration and invasion in PDAC [26]. In this study, we revealed reciprocal suppression between MALAT-1 and miR-200c-3p expression and clarify the underlying mechanism.

Finally, we found that MALAT-1 and ZEB1 was negatively correlated with miR-200c-3p transcript level of PDAC tissues. And there was a positive correlation between MALAT-1 and ZEB1 level. Survival analysis reveals that both MALAT-1 and miR-200c-3p levels are independent prognostic factors. MALAT-1 (high)/ miR-200c-3p (low) correlated with shorter OS for PDAC patients. Interestingly, Li Q et al. [27] showed that MALAT-1 levels were lower in most endometrioid endometrial carcinoma tissues than in normal tissues, while miR-200c-3p levels were higher. Therefore, different expression model of MALAT-1 and miR-200c-3p in human tumor entities needs further mechanism researches.

## Conclusion

**Fig. 10:**
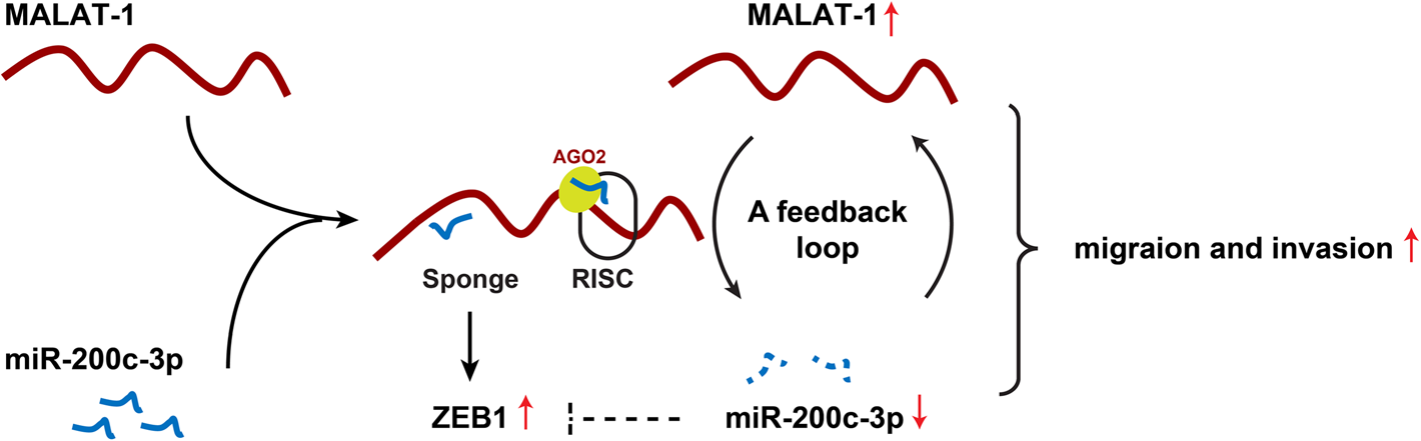
Schematic model of a novel feedback loop between high MALAT-1 and low miR-200c-3p promotes cell migration and invasion in PDAC. MALAT-1 functioned as a miRNA sponge to attenuate the endogenous function of miR-200c-3p, which negatively modulates ZEB1 expression in PDAC. On the other hand, miR-200c-3p inhibited the level of MALAT-1 expression is somewhat similar to the miRNA-mediated silencing of protein-coding genes

In summary, our findings firstly revealed a novel feedback loop between high MALAT-1 and low miR-200c-3p (Fig. 10). MALAT-1 could function as a miRNA sponge to attenuate miR-200c-3p function, leading to ZEB1 upregulation in PDAC. On the other hand, miR-200c-3p inhibited the level of MALAT-1 expression in a way similar to miRNA-mediated silencing of target genes. Targeting the feedback loop between high MALAT-1 and low miR-200c-3p will be a novel therapeutic strategy for PDAC.

## Additional files

Additional file 1: The primer sequences of miR-200c mimic, miR-200c inhibitor, siRNA against MALAT-1, and their respective negative controls. (DOCX 15 kb)

Additional file 2: Initial profiling of miRNAs that have base-pairing with MALAT-1 in response to knockdown of MALAT-1. (DOCX 16 kb)

Additional file 3: Anti-miR-200c-3p restores MALAT-1-siRNA function in PANC-1 and CAPAN-1 cells. a, b. Transwell migration assays were employed to detect cell migration ability when co-transfected PANC-1 (a) and CAPAN-1 (b) cells with MALAT-1 siRNA and miR-200c inhibitor. (TIF 5830 kb)

Additional file 4: Correlation between the clinicopathologic characteristics and MALAT-1 and miR-200c-3p expression in PDAC (*n* = 65). (DOCX 21 kb)

Additional file 5: Summary of univariate and multivariate COX regression analysis of OS duration in all PDAC patients (*n* = 65). (DOCX 20 kb)

## Abbreviations

FBS: Fetal bovine serum
HPDE: Human pancreatic ductal cells
lncRNAs: Long non-coding RNAs
LSD: Least-significant difference
MALAT-1: Metastasis-associated lung adenocarcinoma transcript 1
miRNAs: Micro-RNAs
ncRNAs: Non-coding RNAs
OD: Optical density
OS: Overall survival
PDAC: Pancreatic ductal adenocarcinoma
RIP: RNA binding protein immunoprecipitation
RT-qPCR: Reverse transcription quantitative real-time polymerase chain reaction
