# Supplementary material for: A novel feedback loop between high MALAT-1 and low miR-200c-3p promotes cell migration and invasion in pancreatic ductal adenocarcinoma and is predictive of poor prognosis
Source: BMC Cancer. 2018 Oct 23;18:1032. doi: 10.1186/s12885-018-4954-9 (PMC6199802; doi:10.1186/s12885-018-4954-9)
Supplement: Supplementary file 1 — The primer sequences of miR-200c mimic, miR-200c inhibitor, siRNA against MALAT-1, and their respective negative controls. (DOCX 15 kb) [file 12885_2018_4954_MOESM1_ESM.docx]

|  | Sequence | |
| --- | --- | --- |
|  | sense（5'-3'） | antisense（5'-3'） |
| miR-200c-3p inhibitor nc | CAGUACUUUUGUGUAGUACAA |  |
| miR-200c-3p inhibitor | UCCAUCAUUACCCGGCAGUAUUA |  |
| miR-200c-3p mimic nc | UUCUCCGAACGUGUCACGUTT | ACGUGACACGUUCGGAGAATT |
| miR-200c-3p mimic | UAAUACUGCCGGGUAAUGAUGGA | CAUCAUUACCCGGCAGUAUUAUU |
| MALAT-1 nc | UUCUCCGAACGUGUCACGUTT | ACGUGACACGUUCGGAGAATT |
| MALAT-1 si1 | GGUGGUGGUAUUUAGAUAATT | UUAUCUAAAUACCACCACCTT |
| MALAT-1 si2 | GCGUCAUUUAAAGCCUAGUTT | ACUAGGCUUUAAAUGACGCTT |
| MALAT-1 si3 | GGGCUGACAUUAACUACAATT | UUGUAGUUAAUGUCAGCCCTT |
