# Supplementary material for: A novel feedback loop between high MALAT-1 and low miR-200c-3p promotes cell migration and invasion in pancreatic ductal adenocarcinoma and is predictive of poor prognosis
Source: BMC Cancer. 2018 Oct 23;18:1032. doi: 10.1186/s12885-018-4954-9 (PMC6199802; doi:10.1186/s12885-018-4954-9)
Supplement: Supplementary file 2 — Initial profiling of miRNAs that have base-pairing with MALAT-1 in response to knockdown of MALAT-1. (DOCX 16 kb) [file 12885_2018_4954_MOESM2_ESM.docx]

| **Name** | **Target Location** |
| --- | --- |
| hsa-miR-503-5p | chr11:65271855-65271882[+] |
| hsa-miR-197-3p | chr11:65267968-65267989[+]/  chr11:65268051-65268072[+] |
| hsa-miR-92b-3p | chr11:65268089-65268110[+] |
| hsa-miR-28-5p | chr11:65270164-65270185[+] |
| hsa-miR-25-3p | chr11:65268090-65268110[+] |
| hsa-miR-370-3p | chr11:65270629-65270650[+] |
| hsa-miR-149-5p | chr11:65267993-65268015[+] |
| hsa-miR-155-5p | chr11:65267833-65267853[+] |
| hsa-miR-378a-3p | chr11:65267987-65268008[+] |
| hsa-miR-23b-3p | chr11:65267724-65267744[+]/  chr11:65269269-65269291[+] |
| hsa-miR-506-3p | chr11:65270907-65270927[+]/  chr11:65273028-65273048[+] |
| hsa-miR-135b-5p | chr11:65271610-65271630[+] |
| hsa-miR-129-5p | chr11:65273381-65273401[+] |
| hsa-miR-200c-3p | chr11:65268676-65268698[+]/  chr11:65270667-65270690[+] |
| hsa-miR-17-5p | chr11:65270735-65270756[+] |
| hsa-miR-20a-5p | chr11:65270735-65270756[+] |
| hsa-miR-203a | chr11:65270609-65270628[+]/  chr11:65271593-65271614[+] |
| hsa-miR-1 | chr11:65272142-65272163[+]/  chr11:65272363-65272384[+] |
| hsa-miR-23a-3p | chr11:65267724-65267744[+] |
| hsa-miR-181c-5p | chr11:65270030-65270051[+] |
