# Supplementary figures and images for: A novel feedback loop between high MALAT-1 and low miR-200c-3p promotes cell migration and invasion in pancreatic ductal adenocarcinoma and is predictive of poor prognosis
Source: BMC Cancer. 2018 Oct 23;18:1032. doi: 10.1186/s12885-018-4954-9 (PMC6199802; doi:10.1186/s12885-018-4954-9)

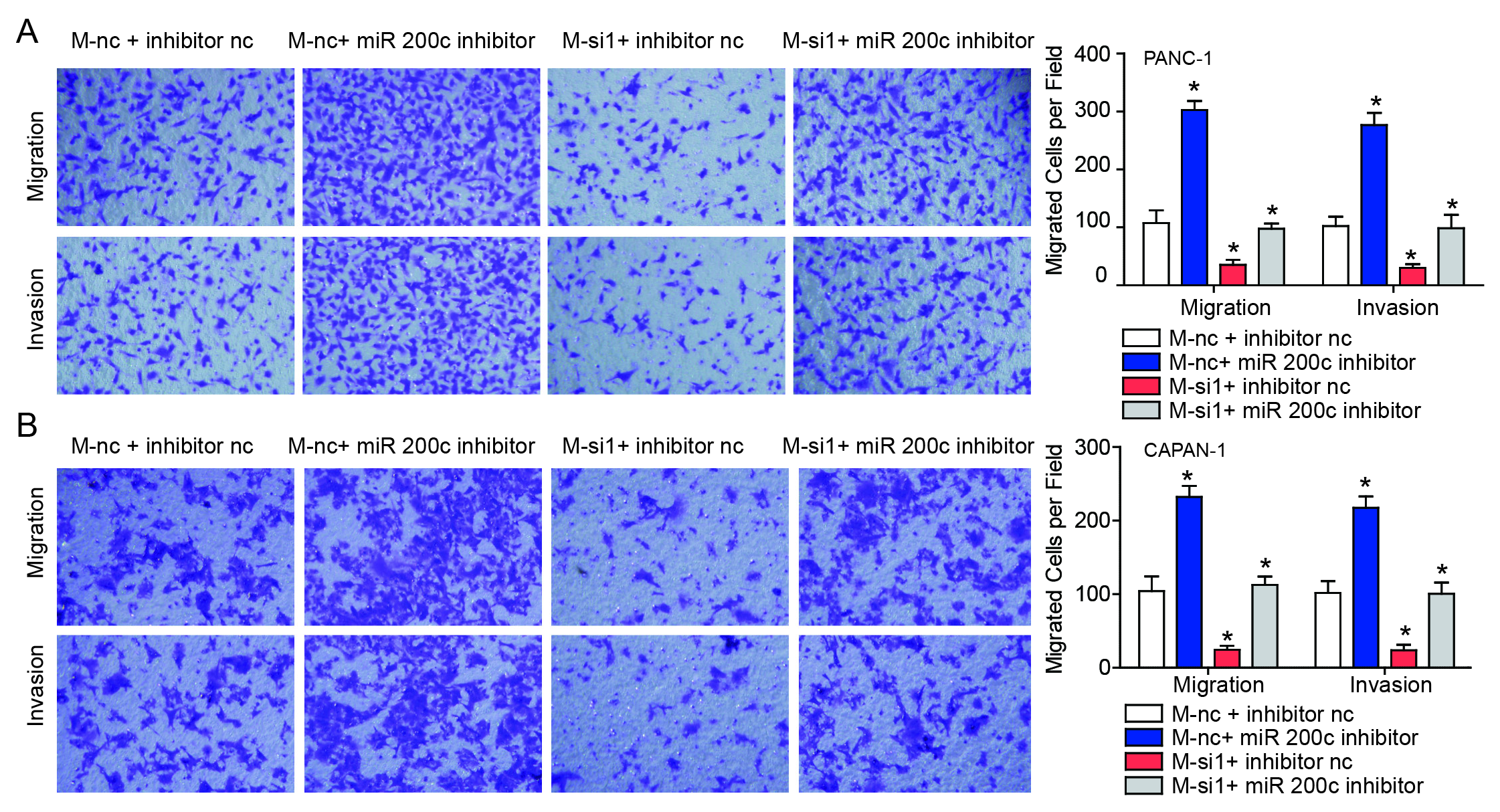

Supplement: Supplementary file 3 — Anti-miR-200c-3p restores MALAT-1-siRNA function in PANC-1 and CAPAN-1 cells. a, b. Transwell migration assays were employed to detect cell migration ability when co-transfected PANC-1 (a) and CAPAN-1 (b) cells with MALAT-1 siRNA and miR-200c inhibitor. (TIF 5830 kb) [file 12885_2018_4954_MOESM3_ESM.tif]
