# Supplementary material for: A novel feedback loop between high MALAT-1 and low miR-200c-3p promotes cell migration and invasion in pancreatic ductal adenocarcinoma and is predictive of poor prognosis
Source: BMC Cancer. 2018 Oct 23;18:1032. doi: 10.1186/s12885-018-4954-9 (PMC6199802; doi:10.1186/s12885-018-4954-9)
Supplement: Supplementary file 4 — Correlation between the clinicopathologic characteristics and MALAT-1 and miR-200c-3p expression in PDAC (n = 65). (DOCX 21 kb) [file 12885_2018_4954_MOESM4_ESM.docx]

| Clinicopathological parameters | No.of patients | MALAT-1 | | |  | miR-200c-3p | | |
| --- | --- | --- | --- | --- | --- | --- | --- | --- |
|  |  | Low | High | *P*-value |  | Low | High | *P*-value |
| Cases | 65 | 33 | 32 |  |  | 33 | 32 |  |
| Age(years) |  |  |  |  |  |  |  |  |
| ≤60 | 25 | 11 | 14 | 0.570^a^ |  | 10 | 15 | 0.170^a^ |
| >60 | 40 | 22 | 18 |  |  | 23 | 17 |  |
| Gender |  |  |  |  |  |  |  |  |
| Male | 43 | 24 | 19 | 0.255^a^ |  | 17 | 26 | 0.011^a*^ |
| Female | 22 | 9 | 13 |  |  | 16 | 6 |  |
| Tumor location |  |  |  |  |  |  |  |  |
| Head, neck | 34 | 19 | 15 | 0.388^a^ |  | 17 | 17 | 0.897^a^ |
| Body, tail | 31 | 14 | 17 |  |  | 16 | 15 |  |
| Tumor size(cm) |  |  |  |  |  |  |  |  |
| ≤3 | 26 | 11 | 15 | 0.265^a^ |  | 12 | 14 | 0.543^a^ |
| >3 | 39 | 22 | 17 |  |  | 21 | 18 |  |
| Tumor differentiation |  |  |  |  |  |  |  |  |
| Well, moderate | 42 | 21 | 21 | 0.867^a^ |  | 21 | 21 | 0.867^a^ |
| Poor | 23 | 12 | 11 |  |  | 12 | 11 |  |
| Invasion depth |  |  |  |  |  |  |  |  |
| T1+T2 | 8 | 3 | 5 | 0.475^b^ |  | 4 | 4 | 1.000^b^ |
| T3+T4 | 57 | 30 | 27 |  |  | 29 | 28 |  |
| Lymph nodes metastasis |  |  |  |  |  |  |  |  |
| N0(negative) | 25 | 18 | 7 | 0.007^a*^ |  | 8 | 17 | 0.017^a*^ |
| N1(positive) | 40 | 15 | 25 |  |  | 25 | 15 |  |
| Distant metastasis |  |  |  |  |  |  |  |  |
| Absent | 62 | 33 | 29 | 0.114^b^ |  | 30 | 32 | 0.238^b^ |
| Present | 3 | 0 | 3 |  |  | 3 | 0 |  |
| Clinical stage |  |  |  |  |  |  |  |  |
| Early stages (≤IIa) | 25 | 18 | 7 | 0.007^a^^*^ |  | 8 | 17 | 0.017^a*^ |
| Advanced stages (>IIa) | 40 | 15 | 25 |  |  | 25 | 15 |  |
| Vascular invasion |  |  |  |  |  |  |  |  |
| Absent | 56 | 26 | 30 | 0.149^b^ |  | 28 | 28 | 1.000^b^ |
| Present | 9 | 7 | 2 |  |  | 5 | 4 |  |
| Nervous invasion |  |  |  |  |  |  |  |  |
| Negative | 23 | 10 | 13 | 0.384^a^ |  | 11 | 12 | 0.725^a^ |
| Positive | 42 | 23 | 19 |  |  | 22 | 20 |  |
| ^a^Chi-square test. ^b^Fisher’s exact test. **P*<0.05 indicates a significant association among the variables. | | | | | | | | |
