# Supplementary material for: A novel feedback loop between high MALAT-1 and low miR-200c-3p promotes cell migration and invasion in pancreatic ductal adenocarcinoma and is predictive of poor prognosis
Source: BMC Cancer. 2018 Oct 23;18:1032. doi: 10.1186/s12885-018-4954-9 (PMC6199802; doi:10.1186/s12885-018-4954-9)
Supplement: Supplementary file 5 — Summary of univariate and multivariate COX regression analysis of OS duration in all PDAC patients (n = 65). (DOCX 20 kb) [file 12885_2018_4954_MOESM5_ESM.docx]

| Clinicopathological parameters | Univariate analysis | | |  | Multivariate analysis | | |
| --- | --- | --- | --- | --- | --- | --- | --- |
|  | HR | 95% CI | P-value |  | HR | 95% CI | P-value |
| MALAT-1 |  |  |  |  |  |  |  |
| Low | 1 |  |  |  | 1 |  |  |
| High | 5.687 | 2.803-11.538 | <0.001* |  | 6.335 | 2.598-15.447 | <0.001* |
| miR-200c-3p |  |  |  |  |  |  |  |
| Low | 1 |  |  |  | 1 |  |  |
| High | 0.175 | 0.083-0.368 | <0.001* |  | 0.294 | 0.133-0.651 | 0.003* |
| Age(years) |  |  |  |  |  |  |  |
| ≤60 | 1 |  |  |  |  |  |  |
| ＞60 | 1.953 | 1.013-3.767 | 0.046* |  |  |  |  |
| Gender |  |  |  |  |  |  |  |
| Male | 1 |  |  |  |  |  |  |
| Female | 1.127 | 0.588-2.161 | 0.718 |  |  |  |  |
| Tumor location |  |  |  |  |  |  |  |
| Head, neck | 1 |  |  |  |  |  |  |
| Body, tail | 1.017 | 0.545-1.898 | 0.959 |  |  |  |  |
| Tumor size(cm) |  |  |  |  |  |  |  |
| ≤3 | 1 |  |  |  |  |  |  |
| ＞3 | 0.872 | 0.469-1.619 | 0.872 |  |  |  |  |
| Tumor differentiation |  |  |  |  |  |  |  |
| Well, moderate | 1 |  |  |  |  |  |  |
| Poor | 0.747 | 0.380-1.469 | 0.398 |  |  |  |  |
| Invasion depth |  |  |  |  |  |  |  |
| T1+T2 | 1 |  |  |  |  |  |  |
| T3+T4 | 0.623 | 0.275-1.414 | 0.258 |  |  |  |  |
| Lymph nodes metastasis | |  |  |  |  |  |  |
| N0(negative) | 1 |  |  |  |  |  |  |
| N1(positive) | 1.826 | 0.946-3.524 | 0.073 |  |  |  |  |
| Distant metastasis |  |  |  |  |  |  |  |
| Absent | 1 |  |  |  |  |  |  |
| Present | 2.402 | 0.549-10.505 | 0.244 |  |  |  |  |
| Clinical stage |  |  |  |  |  |  |  |
| Early stages (≤IIa) | 1 |  |  |  | 1 |  |  |
| Advanced stages (＞IIa) | 2.295 | 1.141-4.616 | 0.020* |  | 3.177 | 1.503-6.716 | 0.002* |
| Vascular invasion |  |  |  |  |  |  |  |
| Absent | 1 |  |  |  |  |  |  |
| Present | 0.904 | 0.377-2.169 | 0.821 |  |  |  |  |
| Nervous invasion |  |  |  |  |  |  |  |
| Negative | 1 |  |  |  |  |  |  |
| Positive | 0.793 | 0.418-1.506 | 0.478 |  |  |  |  |
| HR hazard ratio, 95% CI 95% confidence interval | | | | | | | |
